# Transmission Dynamics and Control of the 2025 Lumpy Skin Disease Epidemic in Sardinia (Italy): A Spatial and Epidemiological Analysis

**DOI:** 10.3390/v18060668

**Published:** 2026-06-12

**Authors:** Federica Loi, Gaia Muroni, Guido Di Donato, Paolo Calistri, Daria Di Sabatino, Stefano Cappai

**Affiliations:** 1 Osservatorio Epidemiologico Veterinario Regionale, Istituto Zooprofilattico Sperimentale della Sardegna, 09125 Cagliari, Italy; gaia.muroni@izs-sardegna.it (G.M.); stefano.cappai@izs-sardegna.it (S.C.); 2 National and OIE Reference Laboratory for Brucellosis, Istituto Zooprofilattico Sperimentale dell’Abruzzo e del Molise ‘G. Caporale’, 64100 Teramo, Italy; gu.didonato@izs.it; 3 National Reference Centre for Veterinary Epidemiology, Programming, Information and Risk Analysis (COVEPI), Istituto Zooprofilattico Sperimentale dell’Abruzzo e del Molise ‘G. Caporale’, 64100 Teramo, Italy; p.calistri@izs.it (P.C.); d.disabatino@izs.it (D.D.S.)

**Keywords:** lumpy skin disease, infectious disease, cluster analysis, epidemiology, basic reproduction number, vaccination efficacy, control strategy

## Abstract

Lumpy skin disease (LSD), a vector-borne viral disease of cattle, re-emerged in Italy in June 2025 after six years of absence in Europe, affecting the island of Sardinia, which had previously been disease-free. The insular setting, the predominance of extensive cattle farming systems, and the rapid implementation of control measures provided a unique opportunity to investigate epidemic dynamics and evaluate vaccination effectiveness under field conditions. This study aimed to describe the epidemiological pattern of the first epidemic season (June–October 2025), estimate key transmission parameters, and assess vaccination effectiveness at the farm level. Confirmed outbreaks consistent with local transmission and notified between 20 June and 26 October 2025 were analyzed to characterize epidemic transmission dynamics, while vaccination effectiveness was assessed over an extended follow-up period through 31 December 2025. The between-farm basic reproduction number (*R*_0_) was estimated from the early exponential growth phase using log-linear regression and doubling time calculations. Spatio-temporal clustering was assessed using Kulldorff’s scan statistic under a Poisson model, accounting for the population at risk. Vaccination effectiveness was evaluated using a time-dependent Cox proportional hazards model with a 21-day post-vaccination lag. A total of 79 outbreaks were confirmed, of which 68 were consistent with local transmission. Affected farms included a total of 3443 cattle, with morbidity, mortality, and case fatality rates of 14.4%, 7.0%, and 31.1%, respectively. The exponential growth phase lasted four weeks, with an estimated growth rate of 0.366 per week and a doubling time of 1.89 weeks. The estimated *R*_0_ ranged from 1.55 to 1.92, depending on the assumed generation time, indicating moderate but sustained transmission. The median apparent spatial spread velocity was 4.8 km/day. Spatio-temporal analysis identified a single highly significant cluster in the central-eastern area, accounting for approximately 27% of outbreaks (RR = 58.06; *p* < 0.001). Vaccination was associated with a substantial reduction in outbreak risk (HR = 0.18; 95% CI: 0.06–0.51; *p* = 0.001), corresponding to an estimated effectiveness of approximately 82% at the farm level. The 2025 Sardinian epidemic was characterized by moderate transmissibility and strong spatial clustering during the early phase. Rapid implementation of vaccination was associated with a significant reduction in outbreak risk, even under conditions of high infection pressure. The integration of spatio-temporal analyses and time-dependent modeling proved essential to support evidence-based control strategies in newly affected regions.

## 1. Introduction

Lumpy skin disease (LSD) is an infectious disease caused by lumpy skin disease virus (LSDV), a member of the genus *Capripoxvirus* (CaPV) within the family *Poxviridae* [1]. LSDV is a large double-stranded DNA virus that is highly stable, exhibits limited genetic variability, and is antigenically related to sheep pox virus and goat pox virus [2,3]. LSD, originally endemic in many sub-Saharan African countries, began to spread beyond the African continent in recent years [4]. In 2012, the disease spread from the Middle East into southeastern Europe, leading to outbreaks in several countries, including EU Member States such as Greece and Bulgaria, as well as non-EU Balkan countries such as Serbia, North Macedonia, and Albania [5,6,7,8].

The spread of LSD in southeastern Europe prompted a coordinated regional response, including mass vaccination campaigns using live attenuated vaccines (primarily the Neethling strain). These efforts proved highly effective, and by 2018 the epidemic had been successfully contained in affected regions, with no further outbreaks reported in vaccinated areas [9,10].

LSDV is highly resistant to inactivation, capable of surviving for up to 33 days or more in necrotic skin nodules, up to 35 days in desiccated crusts, and at least 18 days in air-dried hides [3,11]. LSD is mechanically transmitted by hematophagous arthropods [12]. Mechanical vectors can transmit the virus from asymptomatic, viremic animals—potentially over distances of up to 5 km, particularly under windy conditions [2,13,14]. The risk is further increased by the abundance of blood-feeding insects and the presence of favorable breeding sites on farms, such as standing water and manure [15,16]. Although these routes play a minor role, transmission can also occur through direct contact with infected animals, as well as via fomites, semen, or the placenta [14,17,18]. The incubation period of LSD is approximately 1–4 weeks. The initial clinical signs include lacrimation and fever, accompanied by enlargement of the subscapular and precrural lymph nodes. The disease tends to be more severe in cattle at peak lactation, often leading to a significant reduction in milk production due to high fever, viral infection, and secondary mastitis. Mortality is generally low. In severe cases, ulcerative lesions may develop on the mucous membranes of the eyes, mouth, and nasal cavities, leading to excessive salivation, lacrimation, and nasal discharge. These secretions can contain LSDV and serve as a source of viral transmission. Pox lesions may also affect internal structures such as the pharynx, larynx, trachea, lungs, and various parts of the alimentary tract. Post-mortem examinations in severe cases have revealed pox lesions on the surface of nearly all internal organs [19,20]. Additionally, some infected animals may develop edematous swelling in one or more limbs, resulting in lameness. The diagnosis of LSD involves a combination of clinical, laboratory and epidemiological findings. Clinical signs are not pathognomonic and must be confirmed by laboratory tests. The gold standard for the detection of LSDV DNA in skin nodules, blood and secretions is the Polymerase Chain Reaction (PCR). Enzyme-linked immunosorbent assays (ELISAs) or Virus Neutralization Test (VNT) are widely used to detect antibodies [21].

One of the main risk factors for the introduction of LSD into disease-free areas is the movement of animals from infected regions or countries to disease-free areas [20,22]. LSD typically spreads over relatively short distances, generally between 10 and 20 km, with a propagation rate estimated at up to 2 km per day [16,23,24]. Several studies have shown that the *R*_0_ for LSDV varies significantly by vector species, and estimates depend heavily on factors like the virus’s ability to survive in the environment and the biting rate of insects. Furthermore, *R*_0_ estimations are highest when based on the detection of the virus in skin lesions, intermediate for blood, and lowest when based on blood virus detection [6,16,23]. Vaccination with a live homologous vaccine, when applied as uniformly as possible across the population with high coverage, is the most effective measure to reduce the spread of the LSD virus [9,25]. Six years after the last notification of LSD in the European continent (Russia, 2019 [26]), in June 2025, a new infection was reported in Italy, a previously disease-free country, specifically in the Sardinian island.

This study aims to provide an epidemiological description of the first LSD epidemic season in Sardinia in 2025, including the analysis of outbreak transmission dynamics up to the last detected outbreak (26 October 2025) and the evaluation of control measures implemented through the end of the epidemic response period (December 2025). Furthermore, the study focuses on the main epidemiological parameters describing the spread of the disease across the island, as well as the estimation of the effectiveness of the control measures applied during the study period.

## 2. Materials and Methods

### 2.1. Study Area

Sardinia is the second-largest island in the Mediterranean Sea, with a total surface area of approximately 24,000 km^2^, and is administratively divided into five provinces (Nuoro, Sassari, Oristano, South Sardinia, and the Metropolitan City of Cagliari), comprising 377 municipalities. Although it is the third-largest Italian region by area, Sardinia has one of the lowest population densities in Italy (approximately 69 inhabitants/km^2^), with extensive rural and sparsely populated areas. About 34% of the territory is devoted to agriculture, nearly 60% to livestock farming, and the remaining portion is covered by woodland. The island has a long-standing agro-pastoral tradition, with sheep and goat farming historically representing the backbone of the regional economy. The bovine sector consists of approximately 286,072 cattle distributed across 8455 farms. Cattle are predominantly reared under extensive or free-ranging conditions, either on private holdings or on public grazing lands, where animals from different owners may share pasture and come into direct contact. Beef production represents the primary objective of cattle farming on the island. Farmers often manage herds across multiple, geographically distant plots, and while the introduction of cattle from outside Sardinia is relatively limited, animal movements within the island are frequent. In addition, a proportion of calves born in Sardinia are routinely exported to mainland Italy for further fattening [27].

### 2.2. Data Collection

The overall study period extended from 20 June to 31 December 2025, corresponding to the epidemic response period. However, different analytical timeframes were applied according to the study objectives. Analyses focused on outbreak occurrence, transmission dynamics, and spatial spread included outbreaks notified from 20 June to 26 October 2025, corresponding to the interval between the first and last confirmed outbreaks. The extended period through 31 December 2025 was used for analyses related to control activities and vaccination effectiveness, allowing complete follow-up after implementation of intervention measures. The data included in this study were collected from official sources: the Italian Veterinary National Database (BDN) and the Veterinary Information Systems of the Italian Ministry of Health (VETINFO). The source database included all registered cattle farms located on the island of Sardinia. From this source population, analytical datasets were constructed according to the objectives of the different analyses. Available variables included location (region, province, municipality, latitude, and longitude), date of suspicion, date of notification (if LSDV infection was confirmed by PCR), farm type (cattle-only or mixed), number of animals present, number of symptomatic animals, number of confirmed cases, number of dead and culled animals, and vaccination data. Specific information on clinical signs and lesions was collected during epidemiological investigations and recorded in the database. All data were stored in Microsoft Excel for Microsoft 365 (Microsoft Corporation, Redmond, WA, USA) using a structured data collection format and were password-protected.

Before proceeding with the statistical analysis, data quality and completeness were assessed through a series of automated and manual validation procedures. Records were checked for duplicate farm identifiers, missing values in key variables (farm code, municipality, geographic coordinates, outbreak dates, and vaccination dates), impossible or inconsistent dates (e.g., vaccination dates preceding farm registration or outbreak notification), and discrepancies between outbreak records and official veterinary information systems. Identified inconsistencies were verified against the original records and corrected when necessary. Missing values were uncommon and mainly involved geographic coordinates (<0.1% of farms). Overall, fewer than 1% of records required manual verification or correction.

Two databases were created. The first dataset (DTB_cases) included all confirmed outbreak events recorded during the study period, each georeferenced at the farm level using latitude and longitude coordinates (WGS84 reference system). For each outbreak, the date of clinical suspicion was used as the event date. The DTB_cases dataset was used to estimate the main epidemiological parameters and perform spatial analyses. The second dataset (DTB_census) included a complete census of all active cattle farms located within the 50 km surveillance area established around the index outbreak following confirmation of the first LSD case in Sardinia. For each farm, geographic coordinates and the number of cattle present were available and included. Although all analytical datasets originated from the same source population, different denominators were used according to the objectives of each analysis. The descriptive epidemiological analysis considered all cattle farms located within the 50 km surveillance zone around the index outbreak (n = 6890). The space–time cluster analysis was restricted to active cattle holdings with at least one animal present within the 20 km protection zone (n = 4474), which represented the area under enhanced active surveillance, as veterinary services were mandated to perform systematic clinical inspections to identify previously undetected outbreaks. In contrast, the vaccination effectiveness analysis included active cattle holdings within the 50 km surveillance zone with complete follow-up information available during the study period (n = 5735). The derivation of these analytical populations is summarized in Table 1.

### 2.3. Population Framework Approach: Full-Population and Locally Transmitted Outbreak Spatial Analysis

**Figure 1:**
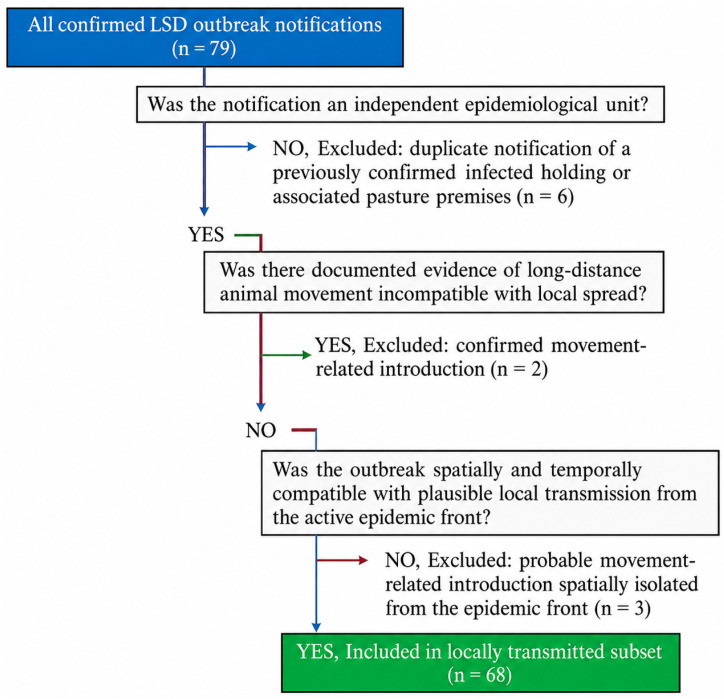
Flow chart of the selection process used to define the locally transmitted outbreak subset.

To disentangle spatial patterns associated with overall outbreak occurrence from those attributable to local transmission, two hierarchical spatial analytical approaches were applied. (a) The full-population analysis quantified the distribution of all notified LSD outbreaks across the entire cattle holding population, capturing both locally generated transmission events and outbreaks resulting from exogenous introductions. This framework provides an operational representation of disease burden and spatial clustering under real-world surveillance conditions. (b) The locally transmitted outbreak analysis focused exclusively on outbreaks deemed consistent with local transmission processes. To accomplish this, the outbreaks were categorized as: (i) outbreaks linked to animal movements based on confirmed introductions from infected farms; (ii) outbreaks probably linked to animal movements based on spatial analysis; and (iii) outbreaks likely attributable to local transmission. Outbreaks considered incompatible with local transmission were excluded based on predefined epidemiological criteria integrating official movement records, epidemiological investigations, and spatial-temporal compatibility assessments. Detailed exclusion criteria and supporting evidence are provided in Figure 1. Farm identifiers and movement record identifiers are not reported for confidentiality reasons. Classification was based on official movement records, epidemiological investigations, and spatial-temporal compatibility with plausible local transmission. This analytical restriction allows inference on geographically structured transmission patterns potentially mediated by proximity-based spread, vector ecology, or local contact networks.

Explorative descriptive statistics were carried out to evaluate the baseline features and their different distribution between the two populations approached (i.e., full-population and locally transmitted outbreaks). Quantitative variables were summarized as mean values, standard deviations (sd), medians and interquartile ranges (IQRs), whereas qualitative variables were summarized as frequencies and percentage. To compare qualitative variables, either the Chi-square test or Fisher’s exact test was applied. To compare differences between quantitative variables, the Kruskal–Wallis nonparametric test was applied. Ninety-five percent confidence intervals for morbidity, mortality, and case fatality rates were calculated using the exact binomial (Clopper–Pearson) method. *p* < 0.05 was considered significant for all analyses, except for multiple comparisons, for which the Bonferroni correction was used. Given the non-random nature of animal movements, exclusion may introduce selection effects. Therefore, comparative and sensitivity analyses were conducted to evaluate the stability of spatial clustering and relative risk estimates under both outbreak classifications. All the statistical analyses described in the following sections were performed separately using both the full-population and locally transmitted outbreak frameworks.

### 2.4. Estimating the Basic Reproduction Number Between Farms

The between-farm *R*_0_ was estimated from the epidemic growth rate during the early exponential phase, under the assumption of an exponential increase in secondary infections [28]. For this purpose, the DTB_cases dataset was organized as a time series including two main variables: the weekly reference date and the number of newly reported outbreaks. Each record corresponded to the number of new outbreaks reported per week, identified by the date of suspicion.

To estimate the *R*_0_, only the initial exponential growth phase of the epidemic curve was considered [29]. Weeks showing a consecutive increase in the number of outbreaks were automatically identified using the first derivative of incidence over time. The start and end weeks of this growth period corresponded to the first and last positive increments in weekly cases, up to the epidemic peak. This semi-automatic step avoided arbitrary selection of the growth phase and ensured consistency across analyses [30,31].

Because weekly outbreak counts represent discrete incidence data, a generalized linear model (GLM) with a log link was fitted to the weekly counts during the exponential phase. Specifically, the model assumed (1):

(1) $$log\left(E\left[I_{t}\right]\right)=rt+\theta$$

where *I_t_* represents the number of new outbreaks in week *t*, *E*[*I_t_*] is the expected weekly incidence, *r* is the exponential growth rate (per week) and *θ* is the intercept. A Poisson error distribution was initially assumed; when overdispersion was detected (dispersion parameter > 1.5), a negative binomial model was fitted instead. The growth rate *r* was estimated by maximum likelihood. The epidemic doubling time ($T_{d}$) was then computed as (2):

(2) $$T_{d}=\;\frac{ln(2)}{r}$$

which represents the number of weeks required for the weekly incidence to double during the exponential phase. Assuming exponential growth and a known mean generation time g (i.e., the average interval between primary and secondary infections at the between-farm level), the *R*_0_ was estimated following the standard relation (3):

(3) $$R_{0}=1+\left(\frac{\ln\left(2\right)\times g}{T_{d}}\right)$$

Uncertainty in parameter estimates was quantified using two approaches. First, 95% confidence intervals (CIs) for r were derived from the Wald approximation based on the model variance–covariance matrix and propagated to *T_d_* and *R*_0_. Second, a parametric bootstrap procedure (5000 replicates) was performed by simulating epidemic trajectories from the fitted GLM (Poisson or negative binomial), refitting the model to each simulated dataset, and recalculating *r*, *T_d_* and *R*_0_. Bootstrap percentile intervals (2.5th–97.5th percentiles) were used as robust 95% CIs.

A sensitivity analysis was performed by varying the assumed mean generation time (*g*) from 1.5 to 4.0 weeks to account for uncertainty in the between-farm generation interval of LSD. The lower range (1.5–2.5 weeks) was selected to reflect the expected interval between infectiousness and detection under intense surveillance and vector-mediated transmission, whereas longer values (3–4 weeks) were included to capture plausible delays associated with the incubation period, between-farm transmission processes, and reporting [20]. For each value of *g*, the corresponding *R*_0_ and its 95% confidence limits were recalculated using both Wald-based and bootstrap-derived uncertainty estimates.

### 2.5. Spatial Analysis

To characterize the apparent spatial progression of the epidemic, outbreaks were chronologically ordered according to the date of clinical suspicion. To assess potential temporal patterns in daily case reporting, daily outbreak counts were first evaluated using the Ljung–Box test to detect autocorrelation over a one-week lag [32]. In addition, a quasi-Poisson generalized linear model with a log link was fitted, with weekday as a categorical predictor to test for overdispersion and potential systematic differences in case counts across days of the week. These analyses were used to identify potential reporting patterns prior to space–time cluster analysis.

Subsequently, pairwise geographic distances between consecutive outbreaks were computed using great-circle distances (Haversine formula), expressed in kilometers. Apparent spatial diffusion velocity was estimated as the ratio between the geographic distance separating two consecutive outbreaks and the corresponding time interval (km/day). Because this metric reflects observed spatial displacement rather than biological transmission speed, it was interpreted as an empirical descriptor of epidemic spread. Given the expected right-skewed distribution due to occasional long-distance transmission events, both mean and median values were reported, with emphasis on the median and interquartile range. To assess the robustness of this descriptive metric in the locally transmitted outbreak subset, a sensitivity analysis was conducted using a nearest-prior-outbreak approach within a seven-day sliding time window. For each locally transmitted outbreak, the geographically nearest outbreak occurring within the preceding seven days was identified, and an alternative apparent diffusion velocity was calculated using the corresponding spatial and temporal separation. This complementary analysis was intended to reduce the influence of temporally interleaved but spatially independent outbreak chains while preserving epidemiologically plausible local transmission linkages. To further assess robustness, the nearest-prior-outbreak approach was also explored across the other analytical subsets for comparative purposes.

To formally assess the presence of statistically significant spatio-temporal clusters during the study period (20 June–31 December 2025) while accounting for the underlying cattle population, Kulldorff’s retrospective space–time scan statistic was applied using the discrete Poisson model [32,33,34]. Under the null hypothesis, outbreaks are assumed to occur randomly over space and time according to a Poisson process proportional to the population at risk, whereas the alternative hypothesis assumes an elevated outbreak rate within at least one scanning window. The scan statistic evaluates cylindrical scanning windows with circular geographic bases of variable radius and temporal heights of variable length. Each cylinder represents a potential space–time cluster. For each candidate window, the observed number of cases within the cylinder is compared with the expected number under the null hypothesis. For each candidate cylinder, a likelihood ratio test compares the observed number of outbreaks inside the scanning window with the expected number under the null hypothesis, and the window with the maximum likelihood ratio is identified as the most likely cluster.

The primary analysis was conducted at the holding level, where each cattle holding represented a spatial unit and each outbreak holding contributed to a single case event defined by the date of outbreak suspicion. The population at risk for the primary space–time scan statistic consisted of active cattle holdings with at least one animal present within the 20 km protection zone. Let *Y_it_* denote the number of outbreak events observed at location *i* and time *t*, and *N_i_* the corresponding number of holdings at risk. Under the null hypothesis, cases are assumed to follow a Poisson distribution $Y_{it}\sim Poisson(\lambda {PN}_{i})$, proportional to the population at risk and constant over space and time, where λ is the baseline incidence rate.

Under this framework, the expected number of outbreak events was assumed to be proportional to the cattle holding population at risk, with a constant baseline incidence over space and time under the null hypothesis. To reduce the influence of the overall epidemic temporal trend, a log-linear temporal trend adjustment with an automatically estimated trend was applied. Statistical significance was evaluated using 999 Monte Carlo replications. Clusters were ranked according to the log-likelihood ratio (LLR), defined as the maximum likelihood ratio comparing the alternative hypothesis (elevated risk within the cylinder) to the null hypothesis. For each statistically significant cluster, the relative risk (RR) was calculated as the ratio of incidence inside the scanning window to incidence outside the window. Only non-overlapping clusters were retained for interpretation. Clusters corresponding to single-farm locations were examined but not interpreted as evidence of spatial diffusion because they did not represent aggregation of multiple outbreak holdings compatible with localized transmission.

All statistical analyses, spatial data processing and exploratory analyses were performed using R software (version 4.4.2, R Foundation for Statistical Computing, Vienna, Austria). The spatial scan statistic was implemented using SaTScan (Boston, MA, USA, version 10.3.3), with geographic coordinates specified in latitude/longitude (WGS84). Mapping outputs were generated using ArcGIS Pro (ESRI, Redlands, CA, USA, version 3.6.3) for contextual visualization.

### 2.6. Space–Time Sensitivity Analysis

Several sensitivity analyses were performed to assess the robustness of the identified spatio-temporal patterns. First, an animal-based Poisson model was fitted using the number of infected animals per farm as case counts and the number of cattle per farm as the population at risk. The cattle population was assumed constant during the study period because movement restrictions prevented substantial changes in herd size. Second, alternative specifications of the spatial and temporal scanning windows were evaluated to assess the sensitivity of cluster detection to model parameterization. Spatial and temporal cluster sizes equal to 10%, 15%, 20%, 30% and 50% of the population and study period, respectively, were explored. Third, the retrospective space–time scan statistic was implemented using the space–time permutation model, which does not require specification of the population at risk.

In addition, a sensitivity analysis was conducted to assess the potential impact of temporal misclassification arising from the use of the date of clinical suspicion as a proxy for the true date of infection or exposure. To evaluate the robustness of the results to this assumption, a stochastic perturbation approach was applied by introducing a random temporal jitter to each outbreak date within a ±seven-day window. This procedure was repeated across multiple simulated datasets, and key epidemiological outputs—including the identification of the exponential growth phase, estimation of the growth rate (*r*) and *R*_0_, and detection of space–time clusters—were compared with those obtained from the original dataset. The aim of this analysis was not to obtain corrected estimates but to verify the stability of the results in terms of order of magnitude and overall epidemiological interpretation.

Finally, to complement likelihood-based cluster detection, an exploratory density-based clustering approach (DBSCAN) was applied to identify areas of high case concentration without distributional assumptions. To evaluate whether identified density clusters could arise under spatial randomness, a permutation-based validation was performed by randomly reallocating spatial coordinates within the study area while preserving the temporal structure of cases (999 simulations). The maximum observed cluster size was compared with the empirical distribution obtained under permutation to derive an empirical significance measure. DBSCAN results were interpreted as complementary to scan statistics and used to assess consistency between density-based aggregations and statistically significant Poisson-based clusters. Concordance was assessed qualitatively through thematic mapping rather than formal statistical comparison.

### 2.7. Testing the LSDV Vaccination Efficacy: Case–Control Framework

The vaccine currently used in Sardinia for the control of LSD in cattle is produced by Onderstepoort Biological Products (OBP), a South African manufacturer. This live attenuated vaccine is based on the LSD Neethling strain, which is the most common strain of LSDV, isolated in Botswana in 1943; it is widely recognized for its efficacy in providing immunity against the disease about one month after inoculation [9,25,35]. It is specifically formulated for use in cattle and is administered via subcutaneous injection at a dose of 2 mL. While the precise viral titer is not publicly disclosed, the vaccine is available in vials containing 25 or 50 doses. Potential side effects are local reactions at the injection site, Neethling disease, temporary decrease in milk production during the first seven days after vaccination.

To evaluate vaccination effectiveness at the farm level, a retrospective cohort design was adopted. All active cattle farms within the 50 km surveillance zone with complete follow-up information available during the study period (n = 5735) were included in the vaccination effectiveness analysis. Farms were followed longitudinally from the start of the epidemic (20 June 2025) until outbreak occurrence or administrative censoring at the end of follow-up (31 December 2025). Administrative follow-up continued through 31 December 2025, including the period after the last confirmed outbreak, to allow adequate observation time for the delayed protective effect of vaccination and completion of control interventions. The outcome of interest was time to the first confirmed LSD outbreak at the farm level. Farms experiencing at least one confirmed outbreak were considered events (outcome = 1), while outbreak-free farms were censored at the end of the observation period (outcome = 0).

Vaccination status was modeled as a time-dependent exposure. For each farm, vaccination was considered effective only after a predefined 21-day post-vaccination lag period, reflecting the time required to develop protective immunity. Farms contributed farm-time as not effectively vaccinated from the beginning of follow-up until the effective vaccination date (vaccination date + 21 days), and as effectively vaccinated thereafter. Consequently, farms vaccinated during follow-up but experiencing an outbreak within the 21-day lag period contributed person-time to the not effectively vaccinated category, reflecting vaccination without expected biological protection rather than the absence of vaccine administration. The primary analysis therefore compared effectively vaccinated farm-time versus not effectively vaccinated farm-time.

Time-to-event analysis was conducted using a Cox proportional hazards model in counting-process form to properly account for time-varying exposure [36]. The hazard of outbreak occurrence for farm *i* at time *t* was modeled as (4):

(4) $$h_{i}\left(t\right)=h_{0}\left(t\right)exp\left(\beta_{i}{Vacc}_{i}\left(t\right)\right)$$

where *h*_0_(*t*) denotes the baseline hazard, *Vacc_i_*(*t*) is the time-dependent vaccination indicator (0 before effective vaccination, 1 after), and *β_i_* is the log hazard ratio associated with effective vaccination. Hazard ratios (HRs) and 95% confidence intervals (CIs) were estimated. Vaccine effectiveness (*VE*) was calculated as (5):

(5) $$VE=\left(1-HR\right)\times 100$$

Vaccination coverage at the farm level was initially explored descriptively. However, according to the vaccination protocol, farms classified as vaccinated were required to achieve at least 95% animal-level coverage. Consequently, farm-level vaccination coverage was highly polarized, with values close to zero among non-vaccinated farms and ≥95% among vaccinated farms. Because of this near-perfect collinearity with vaccination status, vaccination coverage was not included as an additional covariate in the final model. To account for potential spatial heterogeneity in outbreak risk and the non-random implementation of vaccination, an additional sensitivity analysis was performed by implementing a matched nested case–control design. Each locally transmitted outbreak was matched to two control farms selected from the population at risk. Controls were required to be outbreak-free at the date of occurrence of the corresponding case, meaning that farms with an outbreak occurring before the case date were not eligible for selection. This risk-set sampling approach ensured that controls represented farms that were still at risk of becoming a case at the time each outbreak occurred. Farms that remained outbreak-free throughout the study period were also eligible as controls. Matching was based on herd size (number of cattle) and distance from the centroid of the primary space–time cluster. Vaccination status was evaluated at the outbreak date of each matched case and classified as unvaccinated, vaccinated but not yet protected (<21 days after vaccination), or effectively vaccinated (≥21 days after vaccination). Conditional logistic regression was then used to estimate the association between vaccination status and outbreak occurrence within matched sets.

To visually describe outbreak dynamics over time, cumulative incidence functions were derived from the time-dependent Cox model. Because vaccination status changed during follow-up, standard Kaplan–Meier curves based on fixed exposure groups were not appropriate. Instead, vaccination status was treated as a time-varying covariate, and survival curves were estimated using the extended Kaplan–Meier approach derived from the counting-process formulation of the Cox model. Estimated outbreak-free survival probabilities were obtained from the fitted Cox model under two exposure scenarios: (*i*) remaining unvaccinated throughout follow-up and (*ii*) vaccinated and protected from the effective vaccination date onward. Differences between exposure states were assessed using the likelihood ratio test from the Cox regression model. Cumulative hazard functions were also examined to evaluate divergence in risk over time between exposure states.

## 3. Results

The first outbreak of LSD in Sardinia was notified by the Veterinary Services (VS) on 20 June 2025 at a farm located in Orani (Nuoro province). Skin nodules were observed in 16 out of 152 animals. Suspicion of LSD prompted the immediate submission of samples to the National Reference Laboratory for Exotic Diseases (Istituto Zooprofilattico Sperimentale dell’Abruzzo e del Molise), which confirmed the infection and performed genotypisation to trace the source of the outbreak. Subsequently, additional outbreaks were reported, immediately notified and reported in the European Animal Disease Information System (ADIS). In response to the outbreaks, and in accordance with Commission Delegated Regulation (EU) 2020/687, protection zones with a 20 km radius and surveillance zones extending up to 50 km were established. In Sardinia, all animal movements outside the region were suspended, and the affected farms quarantined, and culling of infected animals was initiated. Culling operations started on 26 June 2025. In most cases, the entire cattle population of the affected farm was not immediately slaughtered; priority was given to clinically affected animals due to logistical constraints and pending appeals before the Administrative Court. The remaining animals were vaccinated in accordance with Commission Implementing Regulation (EU) 2023/361 to rapidly reduce viral circulation and prevent further spread of the disease. The final stamping-out operation was carried out on 23 December 2025, resulting in zero remaining infected animals on the island. 

The last outbreak notified during the epidemic season occurred on 26 October 2025; no additional confirmed outbreaks were detected thereafter, although outbreak control activities continued until December 2025. From 20 June to 26 October 2025, LSD caused 79 confirmed outbreaks in farms located in northeastern Sardinia (Nuoro, Sassari, Gallura, and Oristano provinces. Within the 50 km surveillance zone, a total of 6890 farms were present, accounting for 245,223 cattle. Of these farms, 96% (6645) were extensive outdoor cattle farms. Between 20 June and 26 October 2025, a total of 4665 (68%) farms inside the 20 km protection zone were clinically inspected. Across the 79 LSDV-infected farms, 3443 cattle were present, with 348 animals showing clinical signs and 177 deaths. Mortality, morbidity, and case fatality rates of 7.0% (95% CI: 6.7–7.4), 14.4% (95% CI: 13.9–15.0), and 31.1% (95% CI: 29.8–32.2), respectively, were observed. By 31 December 2025, 7277 Sardinian farms were vaccinated, of which 6221 were located within the 50 km surveillance zone. On 23 July, the vaccination campaign started, and approximately 316 farms per week were vaccinated (median = 316; IQR = 15–448). No spatial vaccination strategy was implemented (i.e., risk-based or spatial-based, centrifugal or centripetal), given that the Italian Ministry of Health recommended a full-population contemporary vaccination all over the region. The distribution of vaccinated farms by 15-days time intervals is reported in Figure S1.

### 3.1. Clinical Signs

During LSD outbreaks investigated in the Sardinia region, clinical presentation varied depending on disease stage and infection severity. Early clinical signs included hyperthermia (up to 41 °C), profuse salivation, nasal and ocular discharge, depression, lameness, and reduced feed intake that occasionally progressed to anorexia. Cutaneous nodules were firm and well-circumscribed; in early stages, they were sometimes detectable only by palpation. Lesions initially appeared on the neck, thorax, and perineal region and subsequently spread to other body areas, including the genital region, udder, teats, and limbs. In some animals, nodules coalesced into larger areas of thickened skin. As the disease progressed, nodules enlarged and could ulcerate or develop crusts and necrotic eschars, often becoming painful on palpation. Enlargement of superficial lymph nodes, particularly the popliteal and prescapular nodes, was also observed. Ulcerative lesions were occasionally present on the muzzle, nostrils, and oral mucosa, especially on the hard palate.

**Figure 2:**
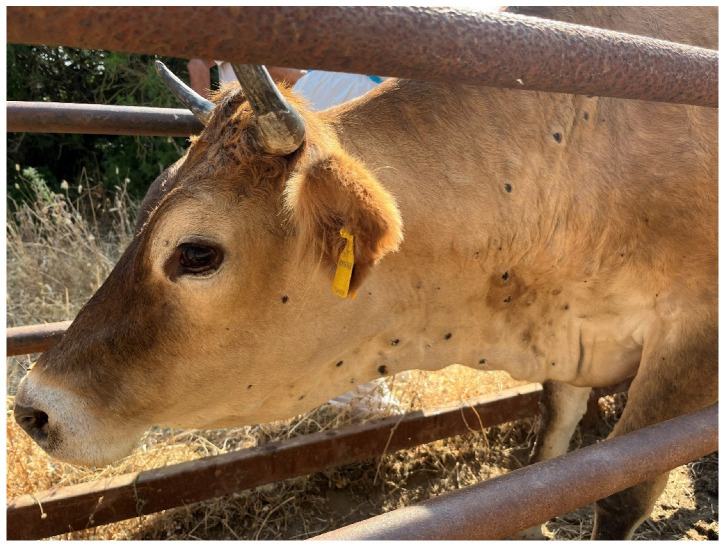
Clinical presentation of lumpy skin disease in cattle. Multiple well-circumscribed cutaneous nodules are visible on the skin surface, consistent with typical lesions observed during field outbreaks.

Post-mortem examination revealed focal intradermal hemorrhages, sometimes in the absence of clinically detectable nodules. Some nodules extended into the dermis, subcutaneous tissue, and occasionally superficial fascia or muscle, with congestion, hemorrhage, and necrosis. Pox-like and ulcerative lesions were also observed in the nasal cavities and trachea. Lymph nodes were typically edematous and occasionally showed diffuse hemorrhages on cut section. Histologically, vascular changes ranged from perivasculitis to necrotizing vasculitis (Figure 2), accompanied by epidermal degeneration, marked mononuclear infiltration in deeper layers, edema, and muscle degeneration, in some cases progressing to myofiber necrosis.

### 3.2. Locally Transmitted Outbreak Selected

**Figure 3:**
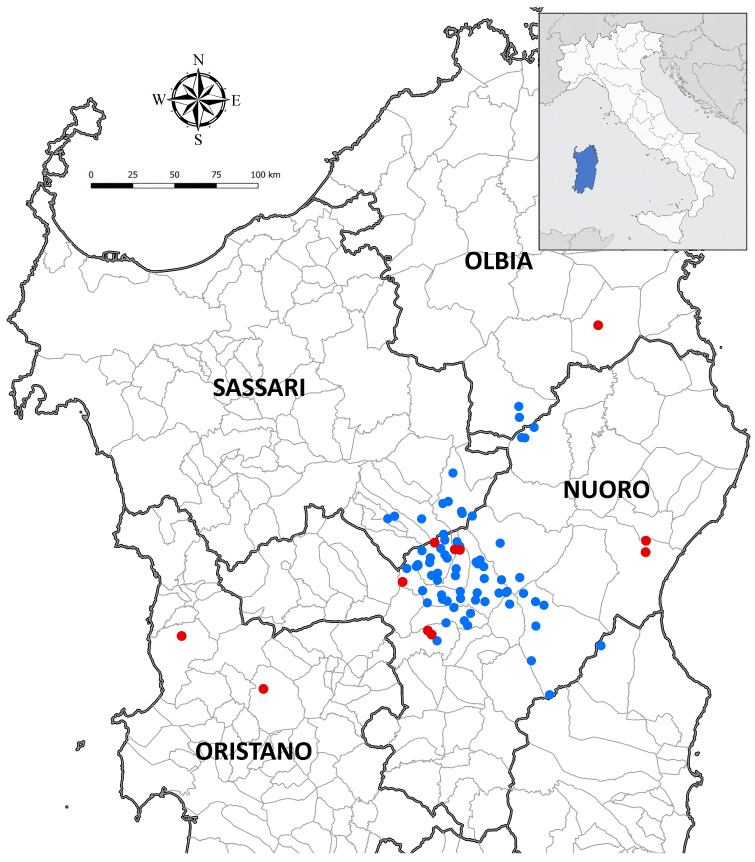
Spatial distribution of outbreak locations (n = 79) in the full dataset. All outbreaks are shown in blue; outbreaks excluded from the locally transmitted subset are highlighted in red (n = 11).

Outbreaks epidemiologically attributed to animal movements were excluded from the analysis of locally transmitted outbreaks. Based on the criteria described above, 11 outbreaks were removed from the dataset as they were classified as certainly or probably linked to animal movements occurring during the period of movement restrictions or identified as spatial outliers. Six outbreaks were excluded because they were identified as duplicate notifications (official movement records showed that animals had been relocated from the notified holding to pasture premises already classified as an outbreak, or vice versa). Five outbreaks were geographically isolated from the main epidemic front and incompatible with plausible local spread dynamics; for two of these, animal movement records retrieved through the T-racing system [37] (movement dates and source/destination holdings) identified epidemiological links with previously infected farms. For the other three, no documented legal movement was identified, suggesting probable unrecorded animal movement. All excluded outbreaks involved domestic cattle and were reported between July and September 2025. As represented in Figure 3, they were located in the provinces of Nuoro (n = 8), Oristano (n = 2), and Olbia (n = 1), and were epidemiologically linked as secondary outbreaks to a previously identified focus. Herd sizes ranged from six to sixty animals, with the number of detected cases per outbreak ranging from two to nineteen. Following the exclusion of these outbreaks, the remaining dataset included 68 outbreaks and was considered representative of outbreaks likely resulting from local transmission processes; it was therefore used for spatial analyses.

### 3.3. Basic Reproduction Number Estimation

**Figure 4:**
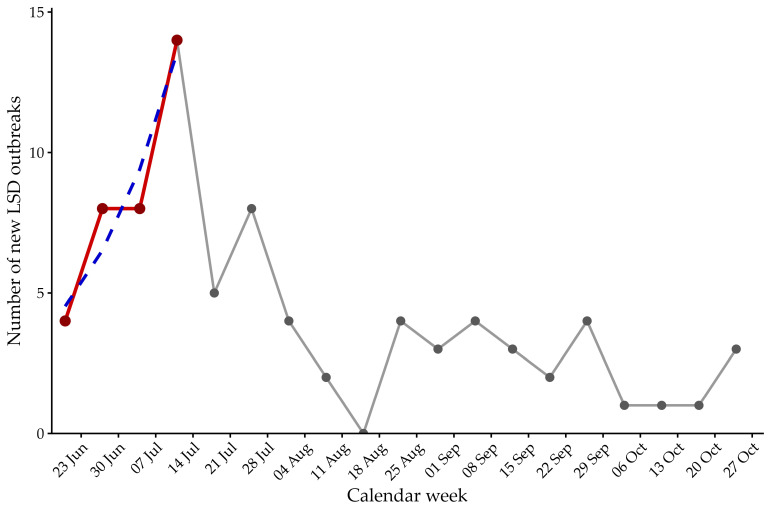
Weekly number of lumpy skin disease outbreaks reported between June and October 2025 (grey line). The red segment indicates the exponential growth phase used to estimate the growth rate (*r*) (20 June to 11 July 2025), doubling time (*T_d_*), and basic reproduction number (*R*_0_). The dashed blue line represents the fitted Poisson model.

The time series datasets (DTB_cases) for the locally transmitted outbreak framework consisted of 19 weekly observations, including 68 outbreaks. The epidemic curve showed an early rapid increase in weekly outbreak counts between late June and early July, followed by a gradual decline until mid-October (Figure 4).

The automated procedure identified the exponential growth period between 20 June and 11 July 2025, corresponding to the first four epidemic weeks, when weekly outbreak counts increased steadily from four to fourteen cases. Beyond this point, incidence values fluctuated and decreased, marking the start of the epidemic decline phase. The estimated mean growth rate (*r*) was 0.366 per week (95% CI: 0.053–0.684, Wald approximation). The corresponding doubling time (*T_d_*) was 1.893 weeks, with a 95% confidence interval of 0.896–9.227 weeks based on bootstrap resampling (Wald CI: 1.013–14.322 weeks). Assuming a mean generation time (*g*) of two weeks, the basic reproduction number was estimated at *R*_0_ = 1.732 (95% bootstrap CI: 1.106–2.451; Wald CI: 1.096–2.367). Sensitivity analysis showed that *R*_0_ ranged from 1.549 (95% bootstrap CI: 1.079–2.008) for *g* = 1.5 weeks to 2.503 (95% bootstrap CI: 1.673–4.071) for *g* = 4 weeks. No evidence of substantial overdispersion was detected in the exponential growth model (residual deviance/df = 0.31); therefore, the final model was fitted using a Poisson error distribution. A summary of the main epidemiological parameters estimated is reported in Table 2.

### 3.4. Spatial Analysis

**Figure 5:**
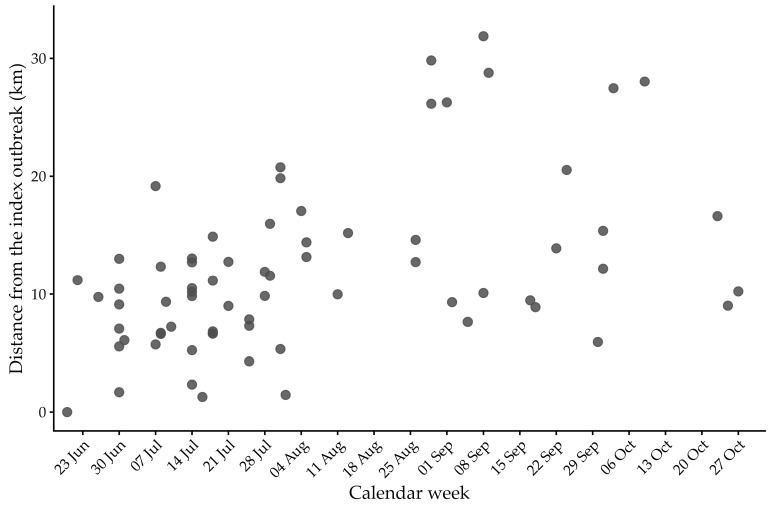
Distance (km) from the first detected outbreak plotted against calendar date, applied to the locally transmitted outbreak population framework.

The 68 locally transmitted outbreaks included were chronologically ordered based on the date of clinical suspicion. The first detected outbreak was used as the spatial reference point for subsequent diffusion analyses. The distance of each outbreak from the index outbreak increased progressively over time, showing a non-linear spatial expansion pattern (Figure 5).

**Figure 6:**
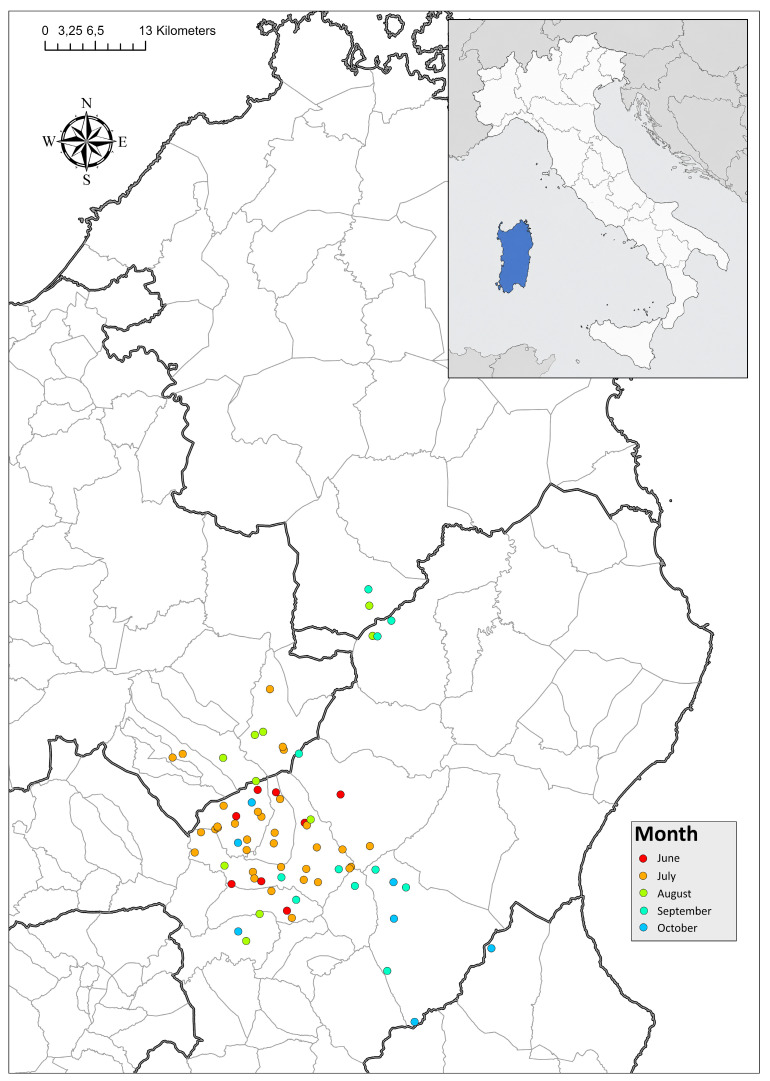
Spatial distribution of LSD outbreaks in Sardinia; gradient colors refer to the month of suspicion.

Early cases were geographically clustered around the index location, followed by a progressive outward spread with occasional long-distance displacement events. The thematic map of outbreaks, colored by date, further illustrates a clear temporal gradient, consistent with spatial propagation from a focal area toward adjacent regions of the island (Figure 6). The pattern suggests an initial spatial concentration followed by radial diffusion with secondary focal aggregations.

**Figure 7:**
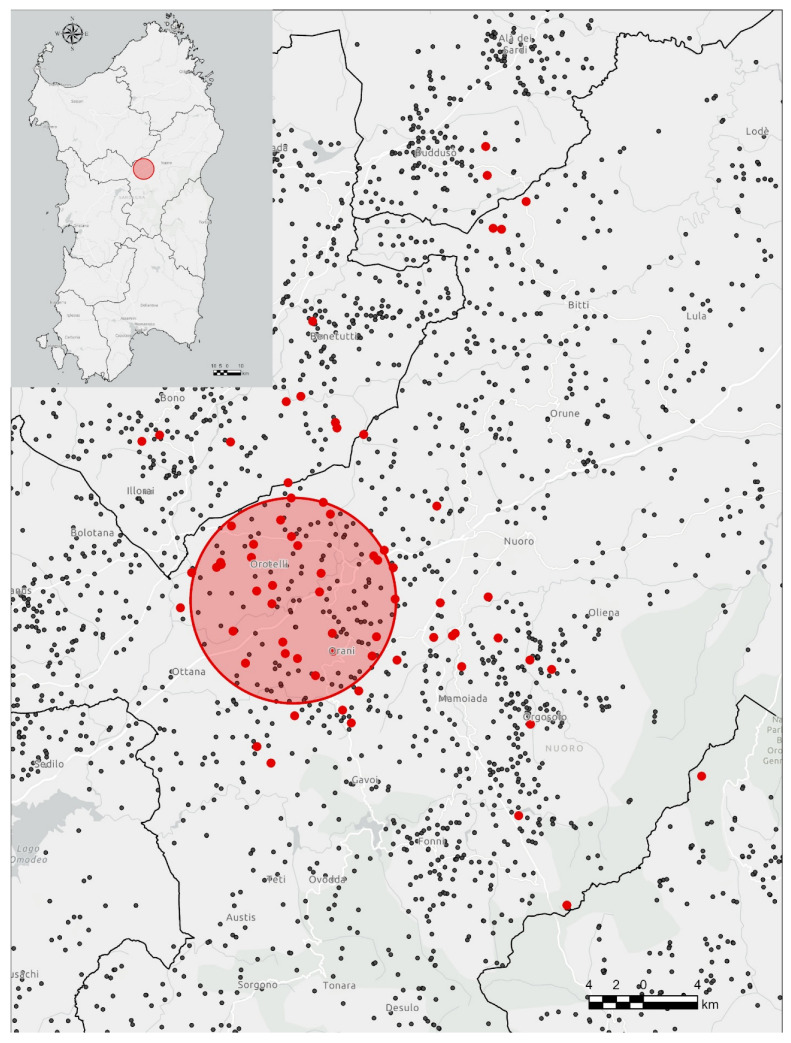
Significant high-rate space–time clusters identified using a retrospective Poisson space–time scan statistic in Sardinia, Italy (2025), representing the location of the most likely high-rate cluster within the region (red buffer), an overview of all holdings (black dots) and lumpy skin disease outbreak holdings (red dots).

A total of 68 outbreak holdings were identified among 4474 active cattle holdings with at least one animal present within the 20 km protection zone during the study period (20 June–31 December 2025) and were included in the space–time cluster analysis. The retrospective space–time scan statistic identified one statistically significant high-rate cluster of LSD outbreaks among cattle holdings during the study period. The most likely cluster was centered at 40.282560 N, 9.134650 E, with a spatial radius of 7.58 km (diameter 15.09 km), and occurred between 30 June and 18 July 2025. The cluster included 142 cattle holdings at risk, among which 19 outbreak holdings were detected during the study period, compared with 0.45 expected under the null hypothesis (observed/expected = 42.12). The estimated relative risk (RR) was 58.06, indicating a markedly elevated risk within the cluster compared with the surrounding area. The cluster was highly statistically significant (log-likelihood ratio (LLR) = 55.34; *p* = 1.3 × 10^−14^, based on 999 Monte Carlo replications). After adjustment for the overall temporal trend (estimated daily decrease of 1.79%), no additional statistically significant clusters were detected, indicating that the observed spatio-temporal aggregation was dominated by a single early epidemic focus. The cluster accounted for approximately 27% of all outbreak holdings detected during the study period (Figure 7).

The median velocity estimated (4.8 km/day) for the locally transmitted outbreak population framework was about four times higher (median = 4.82, IQR = 4.38) than that estimated under the full-population framework, with a minimum value of 0.389 km/day and a maximum value of about 30 km/day. Within the high-rate cluster, the median velocity estimated was equal to 1.4 km/day (IQR = 5.54) (Table 3). To assess the robustness of the locally transmitted outbreak diffusion estimate, an alternative nearest-prior-outbreak approach within a seven-day sliding time window was applied. This sensitivity analysis showed a more conservative median apparent velocity of 1.81 km/day (IQR = 3.06), compared with 4.82 km/day under the consecutive-event approach, suggesting that chronological ordering may overestimate apparent spread when multiple concurrent local transmission chains are present. No statistically significant weekly or daily reporting pattern was detected (Ljung–Box test: X^2^ = 8.87, df = 7, *p* = 0.26), and no significant weekday effect was observed in a quasi-Poisson regression model accounting for overdispersion (F_6_,_32_ = 0.87, *p* = 0.53).

Sensitivity analyses yielded results consistent with the primary holding-level analysis. The animal-based Poisson model, using the number of infected animals per farm as case counts and the number of cattle per farm as the population at risk, identified a spatio-temporal cluster in the same geographic area and time period as the primary holding-level cluster, although with higher relative risk estimates reflecting within-herd transmission intensity. Exploration of alternative spatial and temporal scanning windows (10%, 15%, 20%, 30% and 50%) produced similar spatial patterns, with the primary cluster remaining stable across model specifications. The space–time permutation model, which does not require specification of the population at risk, also detected a significant cluster in the same spatial area during the early epidemic phase, supporting the robustness of the primary findings. The same results were produced when applying the stochastic perturbation approach using a random temporal jitter for each outbreak date within a ±seven-day window. Finally, the DBSCAN density-based clustering approach identified a spatial aggregation of outbreak locations overlapping with the significant Poisson-based cluster. Permutation-based validation indicated that the observed density cluster size exceeded values expected under spatial randomness, further supporting the presence of a localized outbreak focus.

### 3.5. Efficacy of the Vaccination

A total of 5735 farms included in the DTB_census dataset, corresponding to the 50 km surveillance area around the index outbreak, were included in the vaccination effectiveness analysis. During the study period, farms contributed 3177.7 farm-years at risk, over which 68 outbreaks were recorded, corresponding to an overall incidence rate of 2.23 outbreaks per 100 farm-years. When stratified by time-dependent vaccination status, 61 outbreaks occurred during periods classified as non-effectively vaccinated (1407 farm-years at risk), yielding an incidence rate of 4.55 outbreaks per 100 farm-years. In contrast, seven outbreaks occurred during effectively vaccinated periods (1771 farm-years at risk), corresponding to 0.40 outbreaks per 100 farm-years (Table 4). These crude incidence rates indicate a markedly lower outbreak occurrence during periods in which farms were considered effectively vaccinated.

**Figure 8:**
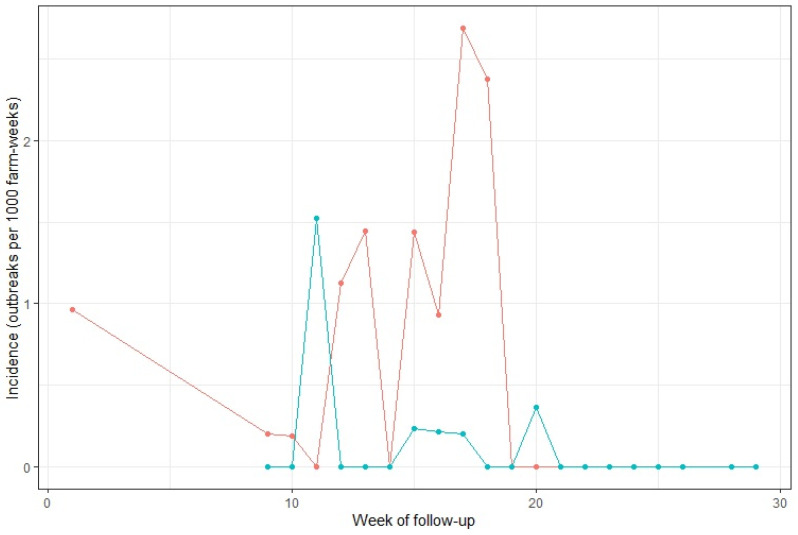
Weekly observed incidence of outbreaks (per 1000 farm-weeks at risk) according to time-dependent vaccination status. The red line represents non-effectively vaccinated farms; the light blue line represents effectively vaccinated farms.

In the time-dependent Cox proportional hazards model, effective vaccination was significantly associated with a reduced hazard of outbreak (HR = 0.18; 95% CI: 0.06–0.51; *p* = 0.001). This corresponds to an estimated 82% reduction in the hazard of outbreak among effectively vaccinated farms compared with non-effectively vaccinated farms. The likelihood ratio, Wald, and score tests were statistically significant (*p* < 0.01). The model concordance was 0.68, indicating moderate discriminative ability, consistent with a model including a single explanatory variable. Results were consistent in the matched nested case–control sensitivity analysis. Effective vaccination remained significantly associated with reduced outbreak risk (OR = 0.25; 95% CI: 0.08–0.80; *p* = 0.02), corresponding to an estimated vaccine effectiveness of approximately 75%. When vaccination status was modeled using three categories, farms vaccinated within the 21-day post-vaccination lag period showed no evidence of protection (OR = 1.79; 95% CI: 0.56–5.73). For effectively vaccinated farms, the estimated odds ratio remained below one (OR = 0.33; 95% CI: 0.09–1.22), although the association was not statistically significant. The weekly observed incidence rates (per 1000 farm-weeks at risk) were consistently lower during effectively vaccinated periods compared with non-effectively vaccinated periods throughout follow-up (Figure 8).

**Figure 9:**
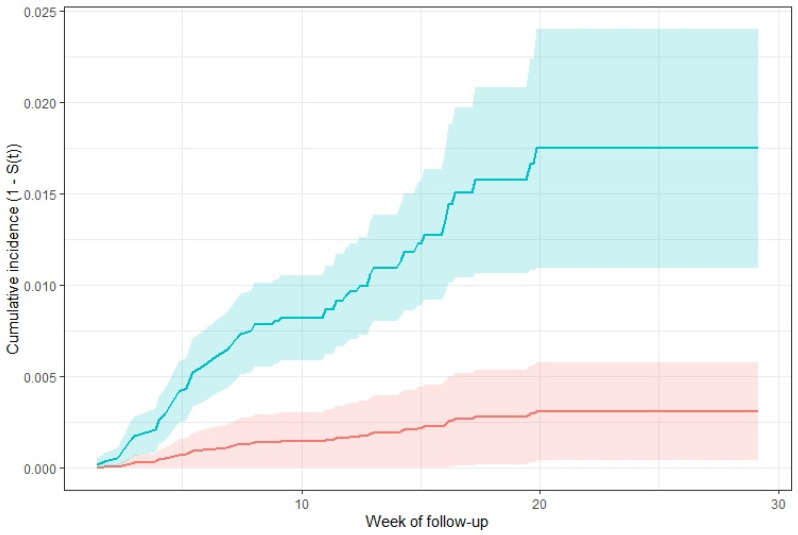
Model-based cumulative incidence derived from the time-dependent Cox proportional hazards model. Shaded areas represent 95% confidence intervals. The red line and boundaries represent non-effectively vaccinated farms; the light blue line and boundaries represent effectively vaccinated farms.

Model-based cumulative incidence curves derived from the Cox model showed clear separation over time. At the end of follow-up, the estimated cumulative incidence was approximately 1.8% among non-effectively vaccinated farms and 0.3% among effectively vaccinated farms (Figure 9). Although the association was statistically significant, the total number of locally transmitted observed outbreaks was limited (n = 68), and the estimates should therefore be interpreted with appropriate caution.

## 4. Discussion

This study provides the first detailed epidemiological characterization of the 2025 LSD epidemic in Sardinia and highlights the main mechanisms that shaped its early spread and subsequent containment. The epidemic showed a short but intense early phase characterized by rapid growth in the number of outbreaks, followed by a progressive decline in incidence after the implementation of control measures, particularly mass vaccination. These results should be interpreted within the specific epidemiological context of Sardinia, where cattle are predominantly raised in extensive grazing systems. In such systems, animals are often distributed across multiple pastures and may share grazing areas, increasing the opportunities for both direct and indirect contacts between herds and potentially facilitating local spread once the virus is introduced.

The space–time analysis identified a single highly significant cluster occurring during the earliest weeks of the epidemic. This cluster largely overlapped with the exponential growth phase used for estimating transmissibility and was geographically concentrated in the area where the first outbreaks were detected. The presence of a dominant early cluster suggests that epidemic expansion was initially driven by a localized transmission focus rather than by multiple independent introductions. Similar spatial patterns have been reported during LSD epidemics in southeastern Europe, where outbreaks tended to cluster around initial foci and spread progressively to neighboring areas [5,8,20,23]. The high log-likelihood ratio and the extremely small Monte Carlo *p*-value further support the presence of a highly structured spatio-temporal process rather than random variation.

The diffusion analysis conducted in this study further supports this interpretation. Most outbreaks were compatible with local spread, while a limited number of cases appeared to reflect long-distance introductions. These events were epidemiologically linked to animal movements and were therefore excluded from the spatial diffusion analysis in order to better capture transmission dynamics compatible with local spread. The importance of uncontrolled animal movements in generating long-distance epidemic jumps has been repeatedly highlighted in LSD epidemiology, particularly during the Balkan epidemics where movement-related introductions occasionally occurred outside the main epidemic front [5,16,20].

The diffusion analysis provided additional insight into the spatial dynamics of the epidemic. Using the original consecutive-event approach, the median apparent propagation velocity among locally transmitted outbreaks was approximately 4.8 km/day, substantially higher than the estimates observed for the full outbreak population (1.8 km/day) and the high-rate cluster (1.4 km/day). However, sensitivity analyses using a nearest-prior-outbreak approach within a seven-day sliding time window yielded a more conservative estimate for the locally transmitted subset (1.8 km/day), while producing only limited changes in the full-population and high-rate cluster estimates. This suggests that the higher value derived from the consecutive-event approach was influenced by the chronological ordering of outbreaks, particularly in the presence of multiple concurrent local transmission chains.

These findings highlight an important methodological distinction between apparent epidemic displacement and biologically plausible transmission speed. The consecutive-event approach captures the observed spatial progression of reported outbreaks but may overestimate transmission velocity when temporally adjacent outbreaks are not directly epidemiologically linked. By contrast, the nearest-prior approach provides a more conservative descriptor of local spread by restricting plausible transmission linkages to geographically proximate outbreaks occurring within a short epidemiologically relevant time window.

The revised estimates are broadly consistent with previously reported LSD spread dynamics. Mercier et al. [5] estimated average epidemic spread rates of approximately 7–8 km/day during the Balkan epidemic, whereas EFSA analyses suggested that most transmission events occurred within distances of 10–20 km, corresponding to substantially lower effective propagation rates under continuous local spread scenarios [20,24]. As also discussed by Gubbins (2019) and Gubbins et al. (2020) [16,23], LSD spatial expansion likely reflects a combination of repeated short-range vector-mediated transmission events and occasional longer-distance dissemination associated with animal movements or other indirect mechanisms.

In the Sardinian context, where cattle are predominantly managed under extensive grazing systems with frequent proximity between neighboring herds, local short-range spread is epidemiologically plausible. At the same time, the island setting and the rapid implementation of movement restrictions likely limited sustained long-distance dissemination, supporting the interpretation of a predominantly focal early epidemic expansion.

The estimated between-farm *R*_0_ during the early epidemic phase was 1.73, assuming a mean generation time of two weeks, indicating that each infected farm generated, on average, approximately 1.7 secondary farm outbreaks during the early epidemic stage. Sensitivity analyses using shorter generation time assumptions (1.5–2.5 weeks) yielded estimates ranging from 1.55 to 1.92. Because the between-farm generation interval for LSD may plausibly exceed the within-farm clinical timeline—particularly when accounting for delays related to incubation, vector-mediated transmission, and outbreak detection—we expanded the sensitivity analysis to include longer mean generation times of three and four weeks. Under these assumptions, the estimated *R*_0_ increased to 2.10 and 2.46, respectively, as expected given the direct dependence of *R*_0_ on the assumed generation interval.

Although these broader assumptions produced higher transmissibility estimates, the overall epidemiological interpretation remained unchanged, supporting sustained but still moderate between-farm transmission during the early epidemic phase. This is consistent with previous field-based estimates for LSD transmission under comparable conditions [9,12]. Importantly, the generation time has a direct linear effect on the estimation of *R*_0_, and uncertainties in this parameter translate into proportional variations in transmissibility estimates. Although the uncertainty around the doubling time was relatively wide—reflecting the limited number of observations in the early growth phase—the bootstrap estimates consistently indicated *R*_0_ > 1. Overall, these results suggest that the epidemic had sufficient transmission potential to sustain spread in the absence of control measures but remained within a range that could be effectively controlled once interventions were implemented. Field estimates of LSD transmissibility are relatively limited, but comparable magnitudes have been reported in previous studies [6,16].

It is important to note that the *R*_0_ estimated in this study reflects transmission dynamics in a very specific production context. Sardinian cattle farming is characterized by extensive grazing systems, where animals spend most of the year outdoors and where physical proximity between herds may occur through neighboring pastures. These conditions may increase the likelihood of mechanical transmission by biting insects, which are considered the main drivers of LSD spread [3,23]. At the same time, the island geography and the rapid implementation of movement restrictions may have limited large-scale dissemination once the epidemic was detected.

Another important result of this study is the estimated effectiveness of vaccination. The time-dependent Cox model showed that effective vaccination was associated with a hazard ratio of 0.18, corresponding to an estimated vaccine effectiveness of approximately 82% at the herd level. This estimate is remarkably consistent with field observations from the Balkan LSD epidemics, where the live attenuated Neethling vaccine demonstrated an effectiveness of approximately 80% in reducing outbreak risk [10]. These findings further support the key role of vaccination as the most effective tool for controlling LSD epidemics in endemic and newly affected regions. In Sardinia, vaccination formed a central component of a broader integrated control strategy, implemented both in response to detected outbreaks and as a preventive measure to reduce the risk of further spread.

The decline in outbreak incidence observed after the vaccination campaign is consistent with previous experiences in Europe, where high vaccination coverage rapidly interrupted LSD transmission [10,24]. Moreover, modeling studies have shown that maintaining high vaccination coverage is essential to reduce the effective reproduction number below the epidemic threshold and prevent further spread [23]. Although other control measures such as stamping-out and movement restrictions likely contributed to epidemic control, the strong association observed between vaccination status and outbreak risk supports the interpretation that vaccination played a major role in reducing transmission during the epidemic. In Sardinia, the control strategy included a combination of stamping-out, mass vaccination, movement restrictions, enhanced biosecurity measures, disinfection procedures, epidemiological investigations, and public awareness activities. The integrated implementation of these measures likely contributed synergistically to the rapid containment of the epidemic. The implementation of these measures required navigating a complex regulatory framework at both national and European levels. In particular, managing movement derogations represented a significant operational challenge for veterinary authorities, highlighting the need for clear and harmonized guidelines during transboundary animal disease emergencies.

The clinical presentation observed during the Sardinian outbreaks was consistent with the classical clinical picture of LSD, including fever, lymph node enlargement, nasal and ocular discharge, lameness and characteristic nodular skin lesions. However, LSD clinical expression is known to be highly variable, and subclinical or mild infections may occur [9]. This variability may complicate early detection, particularly in extensive systems where animals are dispersed across large grazing areas and frequent inspection of individual animals may be difficult. The observed morbidity and mortality rates were consistent with those reported in previously affected regions. However, the likely occurrence of subclinical or mild infections further supports the need to complement clinical surveillance with laboratory diagnostics.

This study has several strengths. First, it relies on official surveillance, outbreak, and vaccination data collected during a real epidemic under field conditions. Second, it integrates multiple complementary analytical approaches, including epidemic growth analysis, spatial clustering, diffusion analysis and survival modeling of vaccination effectiveness. Third, distinguishing outbreaks linked to animal movements from those compatible with local transmission improves the interpretation of spatial transmission patterns. Finally, the Sardinian epidemic represents a particularly informative case study because it occurred in a geographically bounded island region characterized by extensive cattle production and high final vaccination coverage. In addition, the explicit evaluation of the sensitivity of *R*_0_ to assumptions on the generation time provides further robustness to the interpretation of transmissibility estimates.

Several limitations should nevertheless be acknowledged. The analysis covered only the first epidemic season and therefore does not allow conclusions regarding long-term persistence or possible reintroduction of the virus. In particular, the relatively short study period does not allow assessment of potential epidemic peaks in subsequent seasons. The estimation of *R*_0_ relied on a relatively short exponential growth phase and on assumptions regarding the generation time, which introduces unavoidable uncertainty. In addition, the vaccine effectiveness analysis was observational and may still be influenced by residual confounding factors such as spatial heterogeneity in infection pressure or differences in the timing of vaccination implementation. Because no new confirmed outbreaks occurred after 26 October 2025, transmission analyses were restricted to the active epidemic phase, whereas vaccination effectiveness was evaluated using an extended administrative follow-up through 31 December 2025 to capture the delayed onset of vaccine protection. Furthermore, this study did not include direct measurements of vector abundance, insect control practices, or detailed farm management variables that may influence LSD transmission dynamics. In addition, the temporal analyses relied on the date of suspicion as a proxy for the timing of infection, as the exact date of exposure was not available. Although additional sensitivity analyses incorporating temporal and spatial covariates yielded results consistent with the primary findings, residual confounding cannot be completely excluded. In particular, local variation in infection pressure and operational factors influencing the timing of vaccination may have contributed to the observed associations despite the island-wide implementation of the vaccination campaign and the consistency of the matched sensitivity analyses. This may have introduced uncertainty in the reconstruction of the epidemic timeline, particularly in the estimation of epidemic growth parameters and spatial diffusion patterns. Delays between infection, onset of clinical signs, and reporting may vary across farms and over time, potentially leading to temporal misclassification of outbreak events. Such uncertainty may have influenced the estimation of key epidemiological parameters, including the growth rate and the basic reproduction number, as well as the inferred speed of spatial spread. However, given the relatively acute clinical presentation of LSD and the heightened awareness during the epidemic, delays in detection were likely limited, and the use of the date of suspicion represents a reasonable approximation under field conditions. Nevertheless, this limitation should be considered when interpreting the temporal and spatio-temporal dynamics of the epidemic. Given the relative scarcity of detailed field data on LSD epidemiology, further research is needed to better quantify key parameters and evaluate control strategies under different ecological and production settings.

Despite these limitations, the results provide useful insights into the epidemiology of LSD in an island region characterized by extensive grazing systems. The Sardinian epidemic appears to have been initially driven by a localized transmission focus in a production system where contacts between herds may be relatively frequent due to pasture-based management. At the same time, the moderate estimated transmissibility and the rapid implementation of control measures, including stamping-out policies, movement restrictions and high vaccination coverage, likely contributed to limiting the overall spread of the disease.

Overall, this study highlights the importance of rapid detection, coordinated control measures and high vaccination coverage in controlling LSD outbreaks. In regions such as Sardinia, where cattle are predominantly raised at pasture and opportunities for inter-herd contact may be substantial, large-scale vaccination strategies implemented rapidly across the territory may represent an effective approach to reduce transmission and contain epidemics. The Sardinian experience further underscores the importance of continuous monitoring, strategic vaccination, and effective collaboration between local and international authorities. A coordinated, science-based approach integrating surveillance, vaccination, regulatory frameworks and communication will be essential not only to eradicate LSD from affected regions but also to prevent its reintroduction.

## 5. Conclusions

This study provides a comprehensive epidemiological assessment of the 2025 LSD epidemic in Sardinia, highlighting the key mechanisms underlying its early spread and subsequent containment. The epidemic was characterized by moderate transmissibility, strong spatial clustering during the early phase, and a predominant pattern of local transmission.

Rapid implementation of control measures—particularly mass vaccination—was associated with a substantial reduction in outbreak risk, supporting the effectiveness of vaccination as a cornerstone strategy for LSD control in both endemic and newly affected regions. In extensive grazing systems such as those observed in Sardinia, broad territorial vaccination may represent a robust and practical approach to interrupt transmission.

These findings have important implications beyond the specific study context. LSD is associated with significant economic losses, trade restrictions, and potential impacts on food security, particularly in vulnerable livestock systems. The successful containment of the Sardinian epidemic underscores the value of coordinated, science-based interventions combining vaccination, movement restrictions, and surveillance.

Overall, this study highlights the importance of early detection, rapid response, and high vaccination coverage in controlling LSD outbreaks. Strengthening integrated control strategies and maintaining preparedness will be essential not only to mitigate future epidemics but also to prevent reintroduction in previously disease-free regions.

## Figures and Tables

**Table 1:** Definition and size of the analytical populations used for the descriptive, spatial, and vaccination effectiveness analyses.

Analytical Population	Definition	N Farms	Used For
Source surveillance zone population	All cattle farms located within the 50 km surveillance zone around the index outbreak	6890	Descriptive epidemiology
Active protection zone population	Active cattle holdings with ≥1 animal present within the 20 km protection zone	4474	Space–time scan statistic
Active surveillance zone population	Active cattle holdings with ≥1 animal present and complete vaccination follow-up within the 50 km surveillance zone	5735	Vaccination effectiveness analysis

**Table 2:** Epidemiological parameters estimated for the locally transmitted outbreak population frameworks. Data are presented as estimates (95% confidence intervals).

Parameters					
**Growth rate (*r*)**	0.366 (0.053–0.684)				
**Doubling time (*T_d_*)**	1.893 (0.896–9.227)				
	**Generation Time (*g*)**				
**Basic reproduction number (*R*_0_)**	**1.5 weeks**	**2 weeks**	**2.5 weeks**	**3 weeks**	**4 weeks**
Bootstrap resampling	1.549 (1.079–2.008)	1.732 (1.106–2.451)	1.915 (1.133–2.814)	2.127 (1.294–3.302)	2.503 (1.673–4.071)
Wald approximation	1.549 (1.072–2.025)	1.732 (1.096–2.367)	1.915 (1.121–2.710)	2.103 (1.205–3.298)	2.503 (1.601–3.896)

**Table 3:** Parameter estimates of the velocity of LSD diffusion based on the full population, locally transmitted outbreaks (consecutive and nearest-prior approaches) and the high-rate cluster. Estimates (mean, SD; median, IQR; min-max) are reported in km per day.

	Estimates			
	Mean (SD)	Median (IQR)	Min-Max	Included/ Overall Population
Full population (km/day)	6.66 (10.99)	1.84 (6.28)	0.17–53.01	75/79
Locally transmitted outbreaks—consecutive (km/day)	6.80 (6.76)	4.82 (4.38)	0.40–30.22	39/68
Locally transmitted outbreaks—nearest-prior (km/day)	2.55 (2.26)	1.81 (3.06)	0.02–8.42	65/68
High-rate cluster (km/day)	5.35 (7.77)	1.40 (5.54)	0.53–22.06	17/19

**Table 4:** Descriptive incidence rates of outbreaks according to time-dependent vaccination status among active cattle farms included in the vaccination effectiveness analysis within the 50 km surveillance zone around the index outbreak.

Vaccination Status	Events	Farm-Years at Risk	Incidence (per 100 Farm-Years)
Not effectively vaccinated	61	1407	4.55
Effectively vaccinated	7	1771	0.40

## Data Availability

The farm-level data used in this study contain information derived from official veterinary surveillance systems and are subject to data protection and regulatory restrictions. Aggregated data supporting the findings of this study, together with the R scripts and SaTScan parameter files used for the analyses, are available from the corresponding author upon reasonable request. Access to farm-level data may be granted, subject to authorization by the competent veterinary authorities and applicable data-sharing regulations.

## Supplementary Materials

The following supporting information can be downloaded at: https://www.mdpi.com/article/10.3390/v18060668/s1, Figure S1: Spatial distribution of vaccinated cattle farms by 15-day intervals during the 2025 LSD vaccination campaign in Sardinia. Maps show the location of farms vaccinated within each consecutive 15-day period from the start of the vaccination campaign (23 July 2025) to the end of the study period. Points represent farms receiving vaccination during the corresponding interval and are not cumulative across panels. The red circle indicates the centroid and radius of the primary space–time cluster identified by the scan statistic analysis. The figure illustrates the rapid and geographically widespread implementation of vaccination across the surveillance area, supporting the absence of a targeted spatial vaccination strategy based on outbreak proximity or local infection pressure.
