# Supplementary material for: Transmission Dynamics and Control of the 2025 Lumpy Skin Disease Epidemic in Sardinia (Italy): A Spatial and Epidemiological Analysis
Source: Viruses. 2026 Jun 12;18(6):668. doi: 10.3390/v18060668 (PMC13307606; doi:10.3390/v18060668)
Supplement: Supplementary file 1 [file viruses-18-00668-s001.zip › viruses-4307722-supplementary.pdf]

Supplementary Material

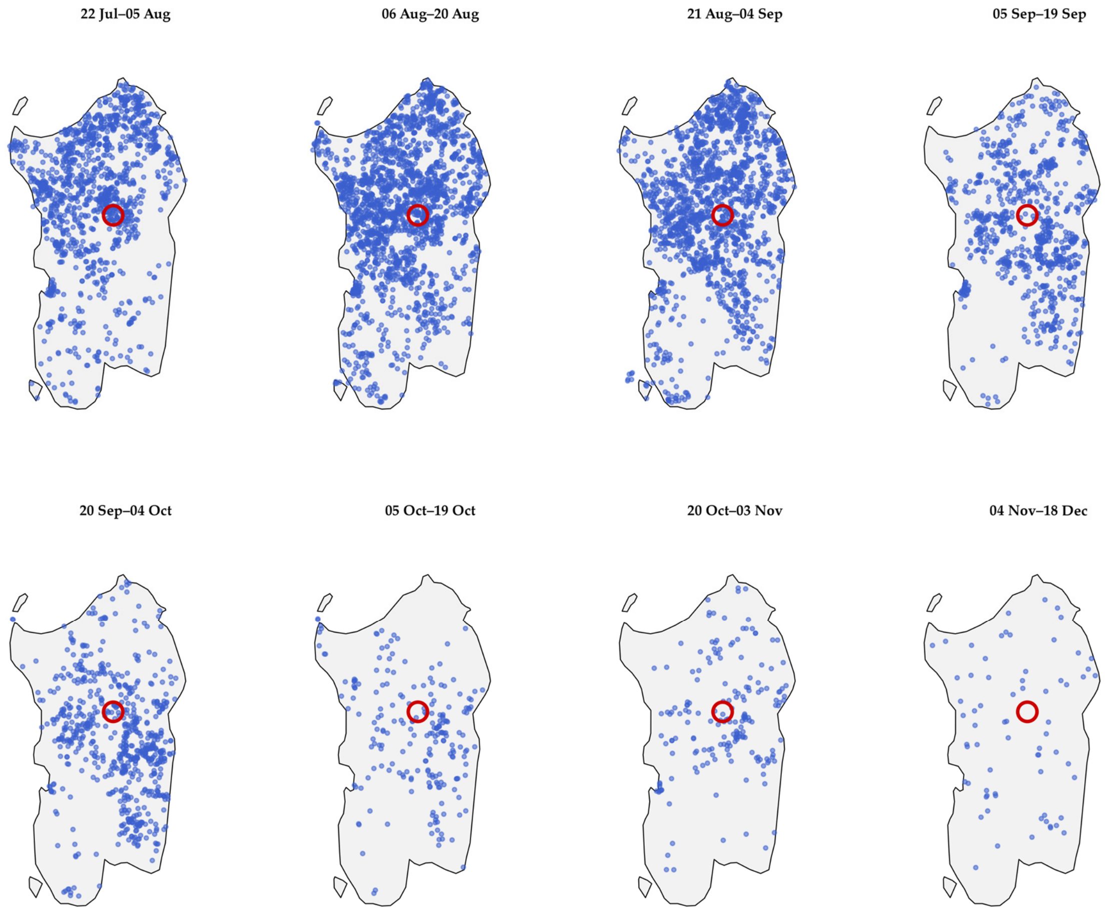

Figure S1. Spatial distribution of vaccinated cattle farms by 15-day intervals during the 2025 LSD vaccination campaign in Sardinia. Maps show the location of farms vaccinated within each consecutive 15-day period from the start of the vaccination campaign (23 July 2025) to the end of the study period. Points represent farms receiving vaccination during the corresponding interval and are not cumulative across panels. The red circle indicates the centroid and radius of the primary space–time cluster identified by the scan statistic analysis. The figure illustrates the rapid and geographically widespread implementation of vaccination across the surveillance area, supporting the absence of a targeted spatial vaccination strategy based on outbreak proximity or local infection pressure.
